# Psychological and Behavioral Adjustment in Patients with Non-Traumatic Lower Limb Amputation and Prosthesis: A Mixed-Method Triangulation Study

**DOI:** 10.3390/jcm14196973

**Published:** 2025-10-01

**Authors:** Marina Maffoni, Alessandra Casati, Clara Tambussi, Valeria Torlaschi, Marco Baldini, Roberto Dragoni, Cira Fundarò, Laura Bagnara, Chiara Ferretti, Antonia Pierobon

**Affiliations:** 1 Psychology Unit of Montescano Institute, Istituti Clinici Scientifici Maugeri IRCCS, 27040 Montescano, Italy; alessandracasati13@gmail.com (A.C.);; 2 Department of Neuromotor Rehabilitation of Montescano Institute, Istituti Clinici Scientifici Maugeri IRCCS, 27040 Montescano, Italy; 3 Neurophysiopathology Unit of Montescano Institute, Istituti Clinici Scientifici Maugeri IRCCS, 27040 Montescano, Italy; 4 Department of Subacute Care of Milano Institute, Istituti Clinici Scientifici Maugeri IRCCS, 20138 Milano, Italy

**Keywords:** lower-limb amputation, vascular diseases, diabetes, amputee, prothesis, mixed-method research, psychological adjustment, rehabilitation

## Abstract

**Background**: Lower limb amputation (LLA), due to non-traumatic causes such as vascular diseases and diabetes, significantly impacts patients’ physical, psychological, and social well-being. While multidisciplinary rehabilitation programs commonly address physical and functional recovery, psychological and subjective experiences related to limb loss remain less explored. Thus, this preliminary study aimed to investigate the psychological and behavioral adaptation processes in patients undergoing rehabilitation following lower limb amputation. **Methods**: A preliminary observational study with a mixed-method approach based on quantitative and qualitative data triangulation was conducted. This approach involves integrating multiple data sources and methodologies—in this case, quantitative psychometric measures and qualitative interviews via the prospective of amputees and those who use prostheses—to enhance the validity and depth of the research findings. **Results**: Fourteen inpatient amputees and fourteen inpatient prosthesis users (years: 66.6 ± 2.5 for amputee and 61.5 ± 1.9 for prosthesis users, male amputees: 85.7%, male prosthesis users: 100%) of a research hospital in the North of Italy were assessed using validated psychometric tools (GAD-7, PHQ-9, PID-5-BF, BIS, ASonA) alongside semi-structured interviews analyzed through the Interpretive Description approach. Key themes highlighted illness acceptance, prosthesis adaptation, body image, medication and behavioral adherence, anxiety, depression, quality of life, denial, optimism, and social support. Overall, anxiety–depressive symptomatology tended to decrease with the prosthesis, and pharmacological and behavioral adherence improved, as did the disease acceptance. Body image was fairly preserved in all patients despite some fears of others’ judgment with respect to the prosthesis. Interestingly, there was poor agreement between quantitative and qualitative data in both the amputee’ and prosthesis users’ groups: while the former returned a partial and neutral picture, a more multifaceted picture emerged from the interviews collected. **Conclusions**: These findings underline the importance of integrating quantitative psychometric evaluations with qualitative methods to comprehensively understand patients’ adaptive experiences. Such combined insights are essential to inform tailored psychological interventions throughout the rehabilitation journey.

## 1. Introduction

Amputation is the surgical removal of all or part of a body segment, typically due to trauma or diseases—such as peripheral artery disease (PAD) or diabetes mellitus (DM)—malignancies, congenital malformations, and chronic infections [1].

DM and PAD remain the primary indications for lower limb amputation (LLA) in high-income countries, confirmed by multiple epidemiological studies [2,3,4,5,6,7,8,9]. In the United States alone, an estimated 150,000 non-traumatic amputations occur annually, predominantly affecting individuals with DM or advanced PAD [2]. In Europe, DM and PAD account for approximately 75–80% of LLAs. Patients with DM are up to 30 times more likely to undergo limb amputation than non-diabetic individuals, with estimates suggesting that one in four diabetic patients will experience LLA during their lifetime [6]. The interplay of peripheral neuropathy, ischemia, and infection constitutes a pathophysiological triad that often culminates in the formation of chronic ulcers and eventual gangrene. Despite notable advancements in diabetic foot care, including screening and ulcer management, LLA rates in this population remain high [7,8,9].

In addition to clinical determinants, several modifiable lifestyle factors significantly increase the risk of LLA. *Smoking* has been consistently associated with impaired peripheral circulation, delayed wound healing, and a higher incidence of major amputations, particularly in patients with diabetes or PAD [10,11]. Likewise, a *sedentary lifestyle* and *unhealthy dietary habits* contribute to metabolic dysregulation and chronic inflammation, exacerbating the risk of diabetic foot complications [12,13]. *Non-adherence* to medical treatment plans—including poor glycemic control, inconsistent foot care, and lack of engagement with screening programs—has also been recognized as a significant contributor to amputation [14,15]. These behavioral risk factors are closely linked to *socioeconomic vulnerability* and *limited health literacy*, underscoring the need for structured patient education, targeted behavior change interventions, and multidisciplinary management strategies tailored to high-risk populations [2,16].

Overall, LLA implies consequences at different levels.

Firstly, it represents a substantial financial burden for healthcare and social systems. Costs include not only the surgical procedure and hospital stay, but also long-term rehabilitation, prosthesis provision, treatment of complications, and social support services. Indirect costs—such as loss of productivity and dependency on caregivers—further amplify the societal impact [17]. These expenses are compounded by frequent re-hospitalizations, prolonged rehabilitation, and psychological interventions required over time [18,19].

Secondly, LLA has profound effects on patients’ emotional lives. Indeed, LLA entails not only the physical loss of a limb, but also the symbolic loss of bodily integrity, autonomy, and identity. Emotional responses range from shock and grief to depression and anxiety, with many patients experiencing a decline in self-esteem and perceived self-efficacy [18]. These outcomes can persist long after surgery, especially in the absence of psychological support [19]. Studies highlight that psychological adjustment to amputation is a dynamic, non-linear process [19,20]. While some individuals exhibit resilience and adaptive functioning, others face prolonged psychological malaise and difficulty in re-establishing daily routines [19]. In this regard, Senra, Oliveira, Leal, and Vieira proposed a three-phase model describing the identity changes experienced by amputees. The first phase involves the initial confrontation with the amputation and coming to terms with the reality of the situation. The second phase encompasses ongoing identity transformations after surgery, affecting various aspects of the self, including social relationships and the acceptance of a new amputee identity, often facilitated by using a prosthesis. The third phase highlights the crucial role of prosthesis in mitigating the negative impacts of limb loss, enhancing the amputee’s physical, psychological, and social well-being, and ultimately improving their quality of life (QoL). Moreover, regarding the subsequent prosthesis use and rehabilitation protocols, the literature shows that patients’ adherence might be influenced by multiple psychosocial variables, including motivation, coping strategies, social support, and satisfaction with the prosthetic device [18,19]. Despite the availability of advanced prosthetic technologies, up to 40–60% of amputees report dissatisfaction and poor compliance, often due to pain, discomfort, or aesthetic concerns [19]. Poor adherence is also associated with higher psychological distress and worse functional recovery [19]. Positive outcomes are more likely when rehabilitation includes psychological counselling, body image restructuring, and strategies to reinforce autonomy and social reintegration [19,21].

However, several critical questions remain unanswered, as this population is still understudied, and there is often inconsistency between findings from quantitative self-report measures and clinical interviews. Bergen and Labonté have emphasized that self-report instruments, such as questionnaires, are highly susceptible to social desirability and self-presentation biases, particularly in the context of stigmatized conditions such as diabetes-related LLA. Consequently, responses may overestimate patients’ QoL, concealing true needs that are more likely to emerge in narrative, interview-based contexts [22].

Given the complexity and the observed discrepancies between qualitative and quantitative findings, triangulation analysis represents a promising approach, as it allows for the parallel examination of diverse data types and the identification of nuances that might otherwise remain concealed. In the field of healthcare services, mixed-methods research is gaining increasing recognition due to its ability to address diverse types of research questions [23]. A systematic review of 168 mixed-methods healthcare studies found that parallel data analysis was the most frequently applied approach, with triangulation serving as a methodological metaphor to support the integration of qualitative and quantitative findings and to foster theory development [24]. Triangulation can deepen the understanding of complex healthcare concepts, such as patient participation, by drawing on multiple data sources and enhancing scientific rigor through discursive processes that challenge interpretations and promote transparency [25]. Thus, this methodological approach benefits both from the narrative richness of the qualitative data and the empirical robustness of the quantitative measurements to achieve a deeper, more comprehensive, and more valid understanding of the phenomenon.

In light of the complex interplay between biomedical, psychological, and economic factors, this study triangulates qualitative and quantitative data from LLA patients to examine how clinical outcomes, emotional adaptation, prosthesis use, and QoL may change. Based on clinical practice, we hypothesize that relying solely on quantitative data may underestimate emotional difficulties or fail to detect certain needs that structured interviews can reveal. The primary objective, therefore, was to develop an integrated profile of psychological functioning in this population by combining quantitative psychometric assessments with qualitative data. Therefore, this triangulation strategy will enhance both validity and reliability, offering a more nuanced and ecologically valid understanding of the rehabilitation process undertaken by this clinical population.

## 2. Materials and Methods

### 2.1. Study Design

The study described in this paper is a preliminary observational study, with no control group or randomization. It adopts a mixed-method approach, and aimed to investigate the psychological and behavioral adjustment processes in individuals undergoing LLA or prosthetic fitting due to vascular and/or diabetic conditions. It is an initial and exploratory investigation. Accordingly, no a priori power analysis was conducted for the quantitative component, and data saturation was not formally assessed for the qualitative interviews.

This study is part of a larger ongoing controlled, randomized, prospective clinical trial at IRCCS Maugeri, Montescano (ClinicalTrials.gov, ID: NCT06471855, 4/4/2025, https://clinicaltrials.gov/study/NCT06471855?cond=NCT06471855&rank=1, accessed on 1 September 2025); Ethical Committee Approval No. 2768, 31/05/2023). This broader trial protocol foresaw the enrolment of 60 participants overall, and detailed in advances all hypotheses and potential outcomes.

The authors employed ChatGPT-4.0 (OpenAI) exclusively for language editing and refinement. All study design, data collection, analysis, interpretation, and manuscript content were conceived and produced independently by the authors, who also carefully reviewed and approved the final text.

### 2.2. Participants

The study included inpatients admitted for multidisciplinary rehabilitation at IRCCS Maugeri. Inclusion criteria were the age ≤80 years and one of the following clinical conditions: (a) being at risk of LLA due to type 2 DM or PAD, (b) being in the post-amputation phase, or (c) undergoing prosthetic fitting after amputation.

In the current paper, triangulation was applied exclusively to data collected from individuals who had already undergone LLA and those in the phase of prosthesis fitting. Indeed, no patients at a high risk of amputation were hospitalized at IRCCS Maugeri during the data collection period. The two groups, although representing different stages within the same clinical pathway, were treated as distinct. All quantitative and qualitative analyses compared amputees and prosthesis users, with the latter’s pre-prosthesis phase excluded to prevent data overlap.

Exclusion criteria included cognitive impairment (Mini-Mental State Examination <26) [26], severe diagnosed medical, psychological or psychiatric conditions, significant musculoskeletal or perceptual deficits that may bias the patient’s response, non-Italian language education, illiteracy, or refusal to participate.

All participants provided informed written consent following ethical standards and privacy regulations.

### 2.3. Instruments

#### 2.3.1. Qualitative Part: Semi-Structured Interviews

Semi-structured interviews were developed based on the Information-Motivation-Strategy Model [27]. It is a theoretical framework designed to understand and predict health-related behaviors. This model posits that health behaviors are determined by three key components: *information* about the health issue, *motivation* to engage in preventive behaviors, and *behavioral skills* necessary to perform these behaviors. Based on these three pillars, the authors developed the interview guide, subsequently thematically divided into three phases:

- *Form A (pre-amputation phase):* explored emotional response, treatment adherence, and quality of life following diagnosis of diabetes or vascular disease;
- *Form B (post-amputation phase)*: addressed psychological impact, stump management, body image, and emotional well-being post-surgery;
- *Form C (prosthesis use phase)*: investigated emotional and functional adjustment to prosthesis, autonomy, and self-image at discharge of a rehabilitation program aiming to adapt to prosthesis.

Each interview concluded with questions regarding the patient’s future perspective and possible perceived identity changes.

#### 2.3.2. Quantitative Part: Psychometric Assessments

Self-report measures were administered to both amputees and prosthesis users. All instruments used in this triangulation study are validated tools widely employed in clinical and rehabilitation settings:

- *EuroQol Visual Analogue Scale (EQ-VAS):*

This is a single-item visual analogue scale assessing perceived health-related quality of life, ranging from 0 (worst imaginable health) to 100 (best imaginable health). Higher scores indicate better perceived health [28,29].

- *Generalized Anxiety Disorder-7 (GAD-7):*

This is a 7-item unidimensional scale measuring the severity of generalized anxiety symptoms over the past two weeks. Each item is rated on a 4-point Likert scale (0 = not at all, 3 = nearly every day). Total scores range from 0 to 21 [30].

- *Patient Health Questionnaire-9 (PHQ-9):*

This is a 9-item scale assessing depressive symptoms according to DSM criteria. Responses are rated on a 4-point Likert scale (0 = not at all, 3 = nearly every day). Total scores range from 0 to 27 [31].

- *Antecedents and Self-Efficacy on Adherence (ASonA):*

This multidimensional questionnaire consists of 20 items and yields both a total score and three subscale scores: Antecedents (ASonA-A), assessing acceptance of health-related limitations, perceived social support, and knowledge of the health condition; Self-efficacy (ASonA-SE), evaluating patients’ self-care strategies and their ability to adhere to both pharmacological treatments (medication intake) and non-pharmacological prescriptions (e.g., physical activity, diet, moderation of alcohol consumption, and smoking avoidance); and Affectivity (ASonA-Aff), which captures the emotional dimensions associated with the health condition. Responses are provided on a 5-point Likert scale, ranging from 1 (strongly disagree) to 5 (strongly agree) [32].

- *Body Image Scale (BIS):*

This is a 10-item unidimensional scale measuring body image distress and dissatisfaction, originally developed for oncological populations but validated for use in amputees. Items are rated on a 4-point Likert scale (0 = not at all, 3 = very much). Total scores range from 0 to 30, with higher scores indicating greater distress [33,34].

At T1, additional instruments were administered to patients undergoing prosthetic adjustment:

- *Trinity Amputation and Prosthesis Experience Scales (TAPES):*

This is a 19-item scale assessing psychosocial adjustment to amputation and prosthesis use, divided into three subscales: psychosocial adjustment (9 items), functional satisfaction (5 items), and phantom limb symptoms (5 items). Items are scored on a 5-point Likert scale (1 = strongly disagree, 5 = strongly agree). Higher scores indicate better adjustment or more symptoms, depending on the subscale [35,36].

### 2.4. Procedure

This study was conducted as a prospective observational mixed-methods design, without randomization or allocation to different treatment arms, as all participants underwent the same standardized rehabilitation protocol, tailored either to the post-amputation phase or to prosthesis adaptation. Upon hospital admission, patients were screened according to the eligibility criteria by a panel of physical and rehabilitation medicine specialists and researchers. All study patients participated in motor rehabilitation (90’) and educational sessions (i.e., nutritional, social, and psychological intervention). Patients were invited to participate during routine psychological consultations. Upon screening for eligibility, they received verbal and written information about the study and provided informed consent. Interviews were audio-recorded and administered according to the patient’s clinical stage, between June 2023 and February 2025. Interview transcripts were subsequently used for qualitative analysis.

All participants also completed the corresponding psychometric tests during hospitalization.

Alongside the research, psychological support was provided to participants in the form of weekly one-hour individual sessions throughout their hospital stay. This support addressed the possible issues and distress that emerged during the interviews, in line with routine clinical care and tailored to the specific needs of each patient.

### 2.5. Data Analysis

To triangulate the data from our sample, quantitative and qualitative analyses were first performed.

For the quantitative analysis, descriptive parameters such as median and measures of normality (e.g., skewness) and frequencies were provided to characterize the two groups: amputees and prosthesis users. Additionally, descriptive statistics, including mean and standard deviation, were calculated for all psychometric measures.

Regarding qualitative analysis, interviews were transcribed verbatim and analyzed using the Interpretive Description (ID) framework [37,38]. This method emphasizes context-specific interpretation and clinical relevance through an in-depth analysis of themes emerging from patient’s narratives. In this case, the focus was put on themes such as emotional impact, self-image, adherence, and expectations for the future. Coding was performed by CA, who had been specifically trained to perform it. Only cases that were uncertain or doubtful were discussed with MM, and further the entire contingency table were reviewed and revised collectively by the entire research team.

Second, to comprehensively explore the multifaceted process of psychological and behavioral adjustment following LLA and prosthesis use, a methodological triangulation approach was employed. This strategy integrates both qualitative and quantitative methods to enhance the depth, reliability, and validity of the findings [23,39]. By integrating distinct data sources—standardized psychometric assessments and semi-structured interviews—the study sought to capture both measurable psychological constructs and the subjective, contextual dimensions of patient experience. Specifically, the use of complementary methodologies—structured self-report questionnaires (quantitative) and interviews (qualitative)—was intended to mitigate the inherent biases associated with each method. While psychometric instruments may be influenced by self-report distortions, interviews conducted within the same clinical context are likewise susceptible to context-related response biases, which warrant explicit acknowledgment. Importantly, the integration of qualitative and quantitative approaches was not conceived as a mere juxtaposition of the two methodologies, but rather as a complementary and interdependent process designed to capture the complexity of the phenomenon under investigation.

Different types of triangulations were identified in the literature [23,39]. In the present study, methodological triangulation was selected, which involves employing multiple research methods (e.g., qualitative and quantitative). This approach is the most commonly used in healthcare and social sciences [23,39]. Specifically, it was implemented by integrating quantitative and qualitative tools to capture both the measurable aspects of psychological functioning and the subjective, narrative dimensions of the lived experience of individuals with amputation and prosthetic use.

From a procedural point of view, the triangulation analysis was applied after each dataset had been analyzed separately: the results from the psychological tests and the interpretations derived from the interviews were first examined independently. This led to the identification of four datasets:

(a) Amputees—Quantitative;
(b) Amputees—Qualitative;
(c) Prosthesis users—Quantitative;
(d) Prosthesis users—Qualitative.

Subsequently, collectively, the authors identified the main themes emerging from the different datasets. This process led to the definition of a list of constructs, referred to as “key findings” in this study, which were then analyzed and compared in terms of presence, frequency, and meaning.

These key findings formed the basis of the Convergence Coding Matrix, a tool used to summarize similarities and differences across datasets. For each identified key finding, comparisons were conducted across the four datasets, with the relationships classified as follows:

- **Agreement** is defined as a complete conceptual alignment between the datasets, where both sources address the same construct with consistent interpretations (e.g., both qualitative themes and quantitative results clearly indicated high levels of distress).
- **Partial Agreement** refers to complementary, but not fully overlapping, findings. This includes cases where one dataset expanded or nuanced the other, or when the alignment was present but not strong or consistent across all participants.
- **Disagreement** is assigned when findings from different sources directly contradicted each other (e.g., a quantitative score indicating high adherence while the qualitative narrative revealed significant treatment avoidance).
- **Silence** indicates that a construct emerged in only one of the two datasets, with no mention or measurable data in the other.
- **Not Applicable** is used when neither dataset contained information related to the construct in question [23].

Dataset comparisons were conducted by CA, with only cases deemed uncertain or ambiguous discussed with MM, followed by consensus meetings involving the other authors to minimize subjective bias and ensure methodological rigor in cross-method validation.

## 3. Results

### 3.1. Sample Characteristics

The sample included in this study comprised 14 adult patients hospitalized after LLA (amputees) and 14 adult patients hospitalized to adapt to a prosthesis (prosthesis users) (Table 1).

The average age of participants was 66.6 years (±2.5) for amputees and 61.5 years (±1.9) for prosthesis users, with the majority being male (amputees: 85.7%, prosthesis users: 100%) and retired (amputees: 50.0%%, prosthesis users: 64.3%). Most patients were single (42.9% both groups); most amputees lived alone (42.9%) and most prosthesis users lived with relatives (35.7%). All participants underwent LLA, primarily at the transtibial level for prosthesis users (100%) and transfemoral level for amputees (42.9%), mainly due to type 2 diabetes mellitus (amputees: 78.6%; prosthesis users: 57.1%).

Regarding health risk behaviors, some subjects reported present smoking habit (amputees: 14.3%, prosthesis users: 28.6%) or having smoked in the past (amputees: 57.1%; prosthesis users: 37.7%). More than half declared not consuming alcohol (amputees: 50.0%; prosthesis users: 57.1%).

Other descriptive data with measures of normality on the two groups are reported in Table 2a.

### 3.2. Quantitative Data

Group comparability was assessed across socio-demographic, clinical, categorical, and ordinal variables. The Shapiro–Wilk test indicated non-normal distributions for some variables; therefore, non-parametric analyses were used. Mann–Whitney U tests showed no significant group differences in age, weight, BMI, years of education, MMSE_PG, EQ-VAS, TOT_GAD, TOT_PHQ, or TOT_BIS (all *p* > 0.05), but a significant difference emerged for height, with the prosthesis group taller on average (U = 54.0, *p* = 0.045). χ^2^/Fisher’s exact tests and ordinal association indices (Gamma, Kendall’s Tau-b) revealed no significant differences or systematic trends for categorical/ordinal variables. Overall, the amputee (A) and prosthesis (P) groups were comparable at baseline, except for height (Table 3).

Regarding cognitive functioning and psychological well-being, amputees and prosthesis users showed similar performance on the MMSE, with average scores of 28.5 (±1.35) and 28.8 (±1.37), respectively. Self-rated health perception (EQ-VAS) was qualitatively higher among prosthesis users (80.4 ± 17.4) than amputees (66.8 ± 21.9). The mean anxiety (GAD-7; amputees: 4.7 ± 5.4, prosthesis users: 4.3 ± 5.8) and depression (PHQ-9, amputees: 4.1 ± 4.6, prosthesis users: 3.6 ± 4.0) remained below the clinical cut-off. Body image dissatisfaction (BIS) scores were comparable between amputees (6.4 ± 6.9) and prosthesis users (6.1 ± 8.6).

All the descriptive data of the above-mentioned quantitative tests (e.g., mean, standard deviation, measures of normality) are reported in Table 4a,b.

### 3.3. Triangulation Data

#### 3.3.1. Comparisons

Data triangulation was performed across four datasets: two qualitative (interviews A/B and C) and two quantitative (psychometric measures for amputees and prosthesis users). Thus, constructs assessed through items of the instruments used (i.e., GAD-7, PHQ-9, EQ-VAS, ASonA, TAPES, BIS) were cross-referenced with qualitative data which were previously analyzed using the Interpretive Description (ID) approach.

**Figure 1:**
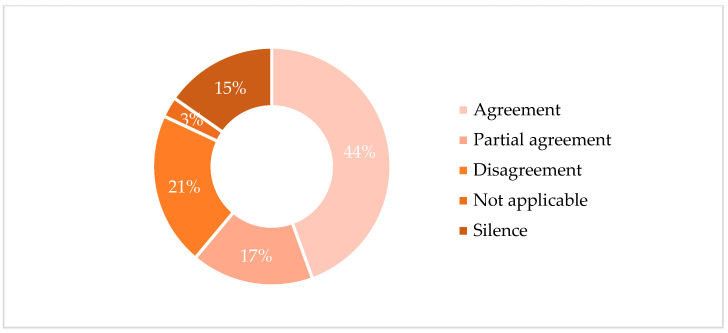
Convergence categories across dataset comparisons.

A total of 12 key findings (below described) emerged from the triangulation. Each key finding was examined through six pairwise comparisons between datasets, resulting in 72 total comparisons (Figure 1): 32 classified as *Agreement*, 12 as *Partial Agreement*, 15 as *Disagreement*, 11 as *Silence*, and 2 as *Not Applicable*.

#### 3.3.2. Key Findings

The 12 key findings identified through data triangulation were organized into four thematic macro-areas: Acceptance and Adaptation, Adherence, Emotional Experience, Social Support (Table A1, Appendix A).

##### Acceptance and Adjustment

*Illness acceptance*

Quantitative data (ASonA) indicated good illness acceptance across both amputees and prosthesis users, with slightly higher scores in the latter. However, qualitative data revealed that amputees expressed a more passive and resigned form of acceptance (*I had to accept it, because there were no alternatives*, pt_020), while prosthesis users linked acceptance to recovery and autonomy (*Now [with prosthesis] I feel normal like everyone else… like there’s nothing different about me*, pt_016).

*Adaptation to the Prosthesis*

TAPES scores showed a high level of adaptation to prosthesis. Interviews confirmed these results with reports of regained independence (*I walked right away… I said ‘guys’, it’s been seven months… I haven’t walked, now I’m going!, pt_016*). However, some patients also reported initial physical fatigue (*I didn’t think it would be so demanding and tiring*, pt_04) and bodily estrangement (*Now I have a piece of body that is not mine…*, pt_05).

*Body Image*

BIS scores were generally favorable across both patient groups, with amputees exhibiting slightly higher values. However, qualitative data revealed significant internal conflicts, particularly among amputees, who frequently reported feelings of incompleteness and concerns about social judgment associated with dissatisfaction regarding their body image (*I want to walk without people noticing I’m missing a limb…*, pt_019). Both, amputee and prosthesis users, complained of phantom limb sensations (*Yes, sometimes… I can feel the limb*, pt_018). Patients with prosthesis also expressed ambivalent concern about their body image (*Sometimes I don’t even feel like looking at myself*, pt_012).

##### Adherence

*Pharmacological and Behavioral Adherence*

Current self-reported adherence from quantitative ASonA scores was high in both groups. However, qualitative narratives contradicted these reports for both groups, revealing prior non-adherence (*I stopped following the therapy… as I had no problems*, pt_05) and persistent unhealthy behaviors (*From time to time I’d take a shot of whisky in the evening*, pt_017). Prosthesis users showed more motivation for change post-intervention (*I want to keep myself more under control now*…, pt_07).

##### Psychological and Emotional Experience

*Anxiety Symptoms*

GAD-7 scores revealed low-level anxiety in amputees and slightly lower scores in prosthesis users. Qualitative data confirmed this, showing mild concerns soon after LLA (*[there is always] the thought of what comes next*, pt_020). Prosthesis users expressed a more positive outlook (*I’m happy, I’m satisfied… it’s fine like this*, pt 010).

*Depressive Symptoms*

Depression scores (PHQ-9) were low among amputees and even lower in prosthesis users; however, qualitative data revealed a contrasting narrative. Both amputees and early-stage prosthesis users reported intense emotional distress, indicating a discrepancy between quantitative measures and subjective experiences (*I’m useless now, what’s the point of me being here?*, pt_018; *I cried for a month… I had no more tears left*, pt_010). Many prosthesis users reported recovery of hope and energy (*Now I see the light at the end of the tunnel…,* pt_01).

*Quality of Life*

EQ-VAS scores were positive overall, especially for prosthesis users. Nevertheless, qualitative data emphasized feelings of diminished autonomy (*What bothers me the most… is not being autonomous in the bathroom*, pt_08). Some improvement was described by prosthesis users post-adaptation (*I mean, at first… it’s difficult, then actually you take two or three steps and… you’re still there, but then you go. I even went up the stairs*, pt_02).

##### Coping and Protective Factors

*Denial*

Although not captured by quantitative measures, denial emerged clearly in the qualitative interviews across both patient groups. Participants frequently minimized the emotional impact of their condition *(“No emotions at all, even when they amputated me…”* (pt_013), and tended to downplay the severity of their situation (*I’ll go back to the way I was… I don’t have a problem*, pt_016).

*Optimism*

Quantitative results (ASonA) revealed medium-to-high optimism, higher in prosthesis users. This was reinforced qualitatively through future-oriented statements (*I’m much more proactive now*, pt_022; *I’m convinced I’ll go back to who I was before*, pt_04).

*Family and Social Support*

Quantitative data from ASonA revealed high levels of perceived familial support in both groups when family is present, with slightly higher values among amputees. Qualitative findings corroborated these results by emphasizing the critical emotional and practical role played by close relatives in the patients’ adaptation processes (*All family around you… give you the strength to move forward, pt_018*). However, social support from friends and broader networks appeared weaker, especially for amputees, who described social isolation and withdrawal linked to physical limitations and fear of judgment (*I’m really making an effort, even just being around people, talking, feeling connected, because I went through a period at home alone, not talking to anyone, always waiting for someone to come over just to have a chat, since I no longer have a car… and then a bit of shame too*, pt_012). Conversely, some prosthesis users reported preserved social engagement (*With friends who keep calling me, asking ‘when are you coming back?*, pt_02).

## 4. Discussion

This paper offers, as far as we know, the first triangulated portraits of psychological and behavioral adjustment in Italian adults with LLA due to PAD and/or DM in a rehabilitation hospital. By systematically cross-examining 72 qualitative–quantitative comparisons, data revealed both convergences and divergences that may be informative for clinical practice. Despite the overall good concordance between quantitative and qualitative findings, comparisons between amputees and prosthesis users revealed important aspects often overlooked by standard questionnaires, such as denial, as well as critical factors frequently underestimated due to response bias, including the true quality of therapeutic adherence and the severity of depressive and anxiety symptoms. These findings support the value of the mixed-method approach as informative in contemporary health–psychology guidelines [40,41].

The demographic and clinical profile of our cohort is consistent with the findings reported in previous studies on LLA populations. Patients were mostly in their sixties, with males representing over 50% of the sample, in line with existing literature [2,3,5,10,19]. Regarding educational background, most individuals had completed lower secondary school, while none had attained a university degree [6]. At the time of recruitment, more than half of the participants were not engaged in employment [18]. For the lifestyle, those who had an LLA reported unhealthy behaviors, including smoking and poor dietary habits, were often influenced by solitary living conditions [15]. The pronounced male predominance and the high proportion of participants who were single or living alone reinforce attention for vulnerable individuals [2].

The key findings of the triangulation analysis deserve various considerations.

First, quantitative data suggest adequate illness acceptance and adjustment in both amputees and prosthesis users, yet interviews disclosed two qualitatively distinct trajectories: a *resigned* acceptance in recent amputees versus a *purpose-driven* acceptance in prosthesis users. These data are in line with previous literature showing that disease and body acceptance go through many challenge phases [19,42,43,44]. This also aligns with Leventhal’s Common-Sense Model of Self-Regulation (CSM), which posits that patients who can effectively link their symptoms, perceived causes, control strategies, and anticipated outcomes are more likely to transition from resignation to active coping [45]. High scores at TAPES and narratives of regained autonomy corroborate the literature reporting that gain in functionality and social activity foster positive acceptance and adjustment [46]. This suggests that the prosthesis serves as a possible salient control cue facilitating adaptive coping. However, residual feelings of bodily estrangement and phantom limb sensations indicate that prosthetic adaptation is not purely mechanical but involves re-negotiation of body schema and identity [20,44]. This issue is relevant in this clinical population as body image is a complex and evolving construct, difficult to assess due to the use of heterogeneous definitions and measurement tools across studies [44]. The integration of the prosthesis into the patient’s body image is strongly influenced by the rehabilitation process, as the restoration of autonomy and functional abilities significantly impacts the extent to which the prosthesis is perceived as part of the self [20,44]. Future research should investigate whether different rehabilitation approaches—traditional versus technologically enhanced—lead to different outcomes in terms of satisfaction and definition of body image, as multisensory feedback in advanced rehabilitation may maximize outcomes [47].

Second, self-report questionnaires indicated high levels of medication and behavioral adherence; however, qualitative interviews revealed instances of prior non-adherence as well as ongoing engagement in risky behaviors (e.g., alcohol habits). Bergen and Labonté suggested that self-report tools, especially in stigmatized conditions such as diabetes-related LLA, are vulnerable to social desirability and self-presentation bias, so questionnaires might be partially misleading [22]. In this regard, triangulating different sources of data can be particularly useful: although each method of collection and analysis of data carries intrinsic limitations, relying solely on standardized tests may overlook important nuances. Clinical experience also shows that in a conversational or interview setting, the narrative mode can elicit subtle aspects of meaning that remain hidden in quantitative scores alone, yet are crucial for designing effective and personalized care paths that address the full range of patient needs. Moreover, from a transtheoretical perspective, the amputation may be a “critical incident” that shifts patients from pre-contemplation to contemplation/action stages of change [48]. Thus, harnessing this crucial moment with motivational interviewing and adherence aids might consolidate healthier routines. Overall, the discrepancy in our data reinforces the value of triangulating self-report with narrative probes and, where feasible, objective markers (e.g., glycemic logs, electronic pill monitors).

Third, concerning anxious and depressive symptoms assessed through GAD-7 and PHQ-9, scores lay in the mild range, apparently contradicting interview narratives of despair, guilt and uselessness. These data may be explained by the fact that there could be response biases underestimating the severity of symptoms measured by self-reported questionnaires [49]. The presence of anxious and depressive symptoms is in line with previous studies: following amputation, certain negative emotional reactions may serve as functional responses to a traumatic event, representing an effort to cope with the adverse condition [50]. It has also been reported that, although depressive and anxiety symptoms are relatively high during the first two years after amputation, they tend to decrease over time, eventually approaching the normal levels observed in the general population [49,50]. Thus, the monitoring of emotional well-being is essential, not only to prevent the development of overt depressive disorders, but also because these emotional factors profoundly influence patients. Emotional distress, feelings of shame, and, in some cases, social withdrawal can indeed have a negative impact on the patient’s quality of life [51]. In our sample, QoL appeared to be closely related not only to the emotions experienced, but, above all, to functional recovery, as patients using prostheses reported higher scores. In turn, the quality of life can both influence and be influenced by rehabilitation outcomes. The literature has highlighted that in amputee patients, traits such as emotional instability and social withdrawal may hinder the rehabilitation process and lower perceived QoL [20,52].

In addition, qualitative data identified denial and minimization as prominent themes, suggesting that patients may understate the psychological impact of their condition by avoiding painful introspection as a protective coping mechanism [19,53]. Thus, denial may serve as a psychological mechanism to alleviate acute emotional distress immediately following surgery; however, if left unaddressed, it may impede the assumption of responsibility for diabetes management, thereby perpetuating the deleterious cycle that ultimately culminated in amputation. Indeed, a systematic review confirms that reliance on avoidant coping predicts poorer psychosocial outcomes in chronic illness and disability [54]. Personality factors, which influence long-term behavior and self-care, warrant greater attention in research on amputee patients [19,53]. Identifying stable personality traits may facilitate the development of personalized interventions aimed at improving treatment adherence. Thus, future studies should include personality assessment, and examine its interplay with socio-demographic, behavioral, and clinical variables. Finally, consistent with emerging research, socio-family support emerged as a pivotal facilitator for patients who underwent LLA, whereas peer and community support were uneven [19,55]. Indeed, social support groups positively influenced emotional well-being and self-efficacy [55]. Therefore, strategic investment in structured social support interventions is essential, especially in settings where family networks are absent or not supportive and for vulnerable individuals who are most likely to experience poorer health outcomes [2].

### Strengths, Limitations, and Future Directions

This study offers several notable strengths. First, its design captures patients’ perspectives at two distinct stages of the rehabilitation pathway (immediately post-amputation and after prosthetic adaptation). Second, the investigation employed a convergence-coding method grounded in established triangulation analysis literature [23,39], enabling the systematic alignment of quantitative indicators with qualitative narratives and uncovering clinically salient constructs that are often overlooked, such as emotional denial.

A limitation concerns the relatively small sample size (28 participants in total, 144 with LLA and 14 completing the prosthetic follow-up). No a priori power analysis was conducted, and the statistical power to detect subtle effects is, therefore, limited. Similarly, for the qualitative component, we did not formally assess data saturation. These features reflect the exploratory and preliminary nature of the study, which aimed to provide an initial triangulated profile of psychological and behavioral adjustment rather than definitive generalizable knowledge. Future research should involve larger samples with formal power calculations and explicit saturation monitoring to ensure robustness and transferability of findings.

The study’s limitations also include the demographic composition of the sample: Italian patients, mainly male, who predominantly live alone. These features limit generalizability to female amputees and cultural contexts with stronger extended-family structures. Moreover, the modest sample size and single-center recruitment may have curtailed variability in socioeconomic status and ethnic background. The limited diversity in clinical settings and participant characteristics underscores the need for future multicenter studies with more heterogeneous cohorts that adequately reflect sex differences, cultural variations, and broader psychosocial conditions.

It should also be noted that the current sample of 28 patients represents the preliminary cohort of a larger clinical trial (NCT06471855), which foresees the enrolment of 60 participants overall. Therefore, the present manuscript reports exploratory findings, while the full protocol will provide sufficient statistical power and broader generalizability.

Another limitation is the absence of a control comparison group of patients who did not receive a prosthesis or who followed alternative rehabilitation pathways. This lack of comparison prevents isolating the specific effect of the prosthesis on psychological adjustment and functional outcomes. Consequently, the observed improvements may partially reflect the natural course of recovery or other rehabilitation components rather than being attributable exclusively to prosthesis use. Future studies should, therefore, incorporate appropriate control groups to strengthen causal inferences regarding the impact of prosthetic rehabilitation.

In addition, the duration of inpatient rehabilitation represents another limitation. In the present study, rehabilitation was restricted to a continuous three-week period. While this timeframe allowed for the observation of short-term adjustments, it provides only a partial view of the long-term psychosocial and functional adaptation process. Future research should, therefore, implement structured follow-up assessments at multiple time points (e.g., six months and one year post-discharge) to evaluate the stability of the observed changes and to capture potential fluctuations in psychological resilience, community reintegration, and prosthetic use over time.

A further limitation is that the type, cost, and technological level of the prostheses were not systematically reported. All devices were standard prostheses provided by the Italian National Health Service, and rehabilitation sessions followed a fixed standardized schedule of 90 min per day. While this choice ensured homogeneity in treatment delivery, it also prevented the exploration of how different prosthetic technologies or rehabilitation intensities might influence satisfaction, adaptation, and long-term outcomes. Future studies should, therefore, stratify patients by type of prosthesis and rehabilitation dosage, to clarify whether technological and training variability may modulate psychological and functional adjustment.

Another methodological limitation concerns the lack of triangulation between functional outcome measures and qualitative data. Although validated functional assessment scales (e.g., Barthel Index, FIM) were available and could provide quantitative information on independence and mobility, the semi-structured interview did not include parallel questions addressing functional status. As a result, it was not possible to systematically integrate or cross-validate these data within the triangulation matrix. Given that emotional recovery is often closely tied to autonomy and mobility, future studies should incorporate qualitative prompts specifically targeting functional domains in order to enable a more comprehensive triangulation between psychological, emotional, and functional outcomes. Moreover, the triangulation was not performed independently and blindly by two raters on the full dataset, so a reliable quantitative measure of agreement between raters is not applicable here. Future research might replicate the triangulation procedure by having it conducted blindly and independently by two or more raters; thus, allowing for the calculation of agreement indices.

Finally, the potential impact of social desirability bias must be considered. While our study acknowledges this bias, we did not reflexively examine how it may have specifically affected our own findings. This issue is particularly relevant given the pronounced discrepancies identified between questionnaire-based scores and interview narratives concerning depression and treatment adherence. The lack of critical self-reflection on this point may have reduced the interpretative strength of our triangulation findings, underscoring the importance of integrating objective measures and reflexive practices in future research.

Future research should, therefore, recruit larger, multicenter cohorts with balanced sex representation, repeated psychological assessments, and objective adherence monitoring. Longitudinal designs that track community reintegration are needed to determine whether the observed transition from resignation to agency is sustained beyond discharge. Finally, randomized trials of body image and peer support interventions, embedded within tailored rehabilitation programs, could translate these findings into healthcare cost-effective strategies that optimize functional outcomes and the quality of life for this growing patient population.

## 5. Conclusions

Psychological adjustment after LLA entails a dynamic process fluctuating between defensive resignation and proactive re-engagement, affected by family resources, prosthetic functionality, and the capacity to integrate a changed body image. Triangulating quantitative scales with qualitative narratives uncovered mismatches—particularly regarding adherence, depression and denial—that are clinically significant. Thus, these preliminary findings suggest their potential impact on clinical practice, encouraging healthcare stakeholders to adopt a multidisciplinary approach in caring for such patients. This should address not only physical and functional needs, but also psychological and relational ones, for example, by implementing group psychoeducational or physiotherapy sessions aimed at normalizing and sharing the LLA experience, thereby helping patients to move beyond defensive resignation toward proactive re-engagement. It is also essential to ensure access to psychological counselling and to promote resources that can support both patients and their families over time, specifically after hospital discharge. Rehabilitation teams adopting this multimodal perspective will be better positioned to detect silent distress, personalize adherence strategies and rehabilitation programs, and foster long-term autonomy, thereby optimizing patient outcomes while reducing healthcare costs.

## Figures and Tables

**Table 1:** Socio-demographical and clinical characteristics of the sample.

	Amputees		Prosthesis Users	
Variable	N	%	N	%
Age				
≤60 years	3	21.4%	5	35.7%
61–70 years	6	42.9%	8	57.1%
71–80 years	4	28.6%	1	7.1%
≥80 years	1	7.1%	0	0%
Gender				
Male	12	85.7%	14	100%
Female	2	14.3%	0	0%
Education level				
Primary school	4	28.6%	4	28.6%
Lower secondary school	6	42.9%	5	35.7%
High school diploma	3	21.4%	5	35.7%
University degree	1	71.1%	0	0%
Postgraduate degree	0	0%	0	0%
Employment status				
Retired	7	50.0%	9	64.3%
Unemployed	1	7.1%	1	7.1%
Employed	6	42.9%	4	28.6%
Marital status				
Single	6	42.9%	6	42.9%
Married	5	14.3%	5	35.7%
Separated	2	28.6%	2	14.3%
Widowed	1	14.3%	1	7.1%
Living situation				
Alone	6	42.9%	3	21.4%
With spouse/partner	2	14.3%	4	28.6%
With partner and sons and daughters	4	28.6%	1	7.1%
With sons and daughters	0	0%	1	7.1%
With other relatives	2	14.3%	5	35.7%
Caregiver				
Spouse	3	21.4%	3	21.4%
Son/daughter	5	35.7%	2	14.3%
Parent	1	7.1%	2	14.3%
Other family member	4	28.6%	4	28.6%
Non-family member	1	7.1%	1	7.1%
None	0	0%	2	14.3%
Level of amputation				
Foot amputation	4	28.6%	0	0%
Transtibial amputation	4	28.6%	14	100%
Transfemoral amputation	6	42.9%	0	0%
Comorbidities				
Type 2 diabetes mellitus	11	78.6%	8	57.1%
Peripheral arterial disease (PAD)	0	0%	1	7.1%
Diabetes + PAD	2	14.3%	5	35.7%
Other	1	7.1%	0	0%
Smoking				
No	4	28.6%	5	35.7%
Yes	2	14.3%	4	28.6%
In the past	8	57.1%	5	35.7%
Alcohol				
No	7	50.0%	8	57.1%
Yes	5	35.7%	3	21.4%
In the past	2	14.3%	3	21.4%

**Table 2:** (**a**) Sample characteristics of amputees with measures of normality. (**b**) Sample characteristics of prosthesis users with measures of normality.

(**a**)												
									**Asymmetry**		**Shapiro-Wilk**	
	**N**	**Missing**	**Average**	**IF**	**Median**	**SD**	**Minimum**	**Maximum**	**Asymmetry**	**IF**	**W**	***p***
Age	14	0	66.64	2.47	65.50	9.24	51	82	−0.116	0.597	0.969	0.857
Weight	14	0	71.64	3.82	73.00	14.31	48.0	95.0	−0.387	0.597	0.924	0.248
Height	14	0	168.64	2.01	166.00	7.52	160	180	0.474	0.597	0.884	0.066
BMI	14	0	25.16	1.14	26.32	4.27	18.6	30.1	−0.415	0.597	0.885	0.069
Education	14	0	9.50	1.27	8.00	4.77	5	23	1.898	0.597	0.759	0.002
Gender	14	0	1.143	0.0971	1.000	0.363	1	2	2.295	0.597	0.428	<0.001
Working status	14	0	2.571	0.5105	1.500	1.910	1	7	0.954	0.597	0.769	0.002
Marital status	14	0	1.857	0.2537	2.000	0.949	1	4	0.951	0.597	0.824	0.010
Living condition	14	0	2.286	0.3841	2.000	1.437	1	5	0.866	0.597	0.813	0.007
Caregiver	14	0	2.714	0.3982	2.000	1.490	1	6	0.734	0.597	0.880	0.058
Comorbidities	14	0	1.500	0.2724	1.000	1.019	1	4	1.781	0.597	0.552	<0.001
Smoke	14	0	1.286	0.2442	2.000	0.914	0	2	−0.662	0.597	0.703	<0.001
Alcohol	14	0	0.714	0.2442	0.500	0.914	0	3	1.368	0.597	0.767	0.002
(**b**)												
									**Asymmetry**		**Shapiro-Wilk**	
	**N**	**Missing**	**Average**	**IF**	**Median**	**SD**	**Minimum**	**Maximum**	**Asymmetry**	**IF**	**W**	***p***
Age	14	0	61.50	1.851	61.50	6.93	50	71	−0.379	0.597	0.944	0.476
Weight	14	0	77.86	2.783	79.00	10.41	60.0	99	0.142	0.597	0.957	0.666
Height	14	0	174.57	1.889	174.00	7.07	161	185	−0.237	0.597	0.970	0.874
BMI	14	0	25.73	1.293	25.45	4.84	18.9	35.9	0.687	0.597	0.954	0.616
Education	14	0	8.86	0.776	8.00	2.91	5	13	0.209	0.597	0.864	0.034
Gender	14	0	1.000	0.0000	1.00	0.000	1	1	Nan	0.597	Nan	Nan
Working status	14	0	1.929	0.3701	1.00	1.385	1	4	0.9580	0.597	0.633	<0.001
Marital status	14	0	1.857	0.2537	2.00	0.949	1	4	0.9507	0.597	0.824	0.010
Living condition	14	0	3.071	0.4504	2.50	1.685	1	5	0.0953	0.597	0.817	0.008
Caregiver	14	0	3.500	0.5522	3.50	2.066	1	7	0.4883	0.597	0.902	0.121
Comorbidities	14	0	1.786	0.2606	1.00	0.975	1	3	0.4921	0.597	0.675	<0.001
Smoke	14	0	1.000	0.2344	1.00	0.877	0	2	0.0000	0.597	0.792	0.004
Alcohol	14	0	0.643	0.2250	0.00	0.842	0	2	0.8287	0.597	0.724	<0.001

**Table 3:** Group comparability data.

Variable	Test	*p*-Value	Effect Size/Association
Age	U = 63.0	0.113	r = −0.357
Weight	U = 73.0	0.259	r = 0.255
Height	U = 54.0	0.045 *	r = 0.449
BMI	U = 96.0	0.946	r = 0.020
Years of education	U = 98.0	1.000	r = 0.000
MMSE	U = 85.0	0.551	r = 0.133
EQ-VAS	U = 69.0	0.187	r = 0.296
GAD-7	U = 87.0	0.625	r = −0.112
PHQ-9	U = 90.0	0.726	r = −0.082
BIS	U = 87.5	0.641	r = −0.107
Gender	χ^2^(1) = 2.15; Fisher = 0.481	0.142	C = 0.267; Tau-b = −0.277
Working status	χ^2^(3) = 1.36; Fisher = 0.838	0.715	C = 0.215; Tau-b = −0.163
Marital status	χ^2^(3) = 0.00; Fisher = 1.000	1.000	C = 0.000; Tau-b = 0.00
Living condition	χ^2^(4) = 5.75; Fisher = 0.244	0.218	C = 0.413; Tau-b = 0.216
Caregiver	χ^2^(5) = 3.62; Fisher = 0.732	0.605	C = 0.338; Tau-b = 0.163
Smoking	χ^2^(2) = 1.47; Fisher = 0.623	0.479	C = 0.223; Tau-b = −0.163
Alcohol	χ^2^(2) = 0.77; Fisher = 0.775	0.682	C = 0.163; Tau-b = −0.023

Note: U = Mann–Whitney U, C = contingency coefficient, Tau-b = Kendall’s Tau-b. * indicates *p* < 0.05.

**Table 4:** (**a**) Descriptive statistics of assessments in amputees. (**b**) Descriptive statistics of assessments in prosthesis users.

**(a)**												
**Descriptive**												
									**Shapiro-Wilk**			
	**N**	**Missing**	**Average**	**IF**	**Median**	**SD**	**Minimum**	**Maximum**	**W**		***p***	
MMSE	14	0	28.500	0.3593	29.000	1.345	26	30	0.889		0.078	
EQ-VAS	14	0	66.786	5.8509	65.000	21.892	20	100	0.968		0.846	
GAD-7	14	0	4.714	1.4428	4.000	5.398	0	17	0.763		0.002	
PHQ-9	14	0	4.071	1.2291	3.500	4.599	0	17	0.812		0.007	
PID-5-BF	14	0	0.514	0.0924	0.540	0.346	0.00	1.32	0.959		0.710	
Adhenrence ASonA	14	0	56.429	3.6969	58.500	13.833	37	76	0.911		0.162	
Self efficacy ASonA	14	0	18.357	1.1222	19.500	4.199	10	24	0.950		0.554	
Affect ASonA	14	0	22.143	2.2795	24.500	8.529	5	32	0.920		0.221	
Total ASonA	14	0	96.929	6.2075	102.500	23.226	65	132	0.921		0.229	
BIS	14	0	6.429	1.8448	4.500	6.903	0	21	0.855		0.026	
(**b**)												
**Descriptive**												
									**Asymmetry**		**Shapiro-Wilk**	
	**N**	**Missing**	**Average**	**IF**	**Median**	**SD**	**Minimum**	**Maximum**	**Asymmetry**	**IF**	**W**	***p***
MMSE	14	0	28.79	0.366	29.00	1.37	26	30	−1.232	0.597	0.796	0.004
EQ-VAS	14	0	80.36	4.643	80.00	17.37	50	100	−0.468	0.597	0.893	0.091
GAD-7	14	0	4.29	1.553	1.50	5.81	0	19	1.518	0.597	0.776	0.003
PHQ-9	14	0	3.57	1.073	2.50	4.01	0	13	1.383	0.597	0.813	0.007
Total BIS	14	0	6.07	2.288	2.00	8.56	0	25	1.557	0.597	0.724	<0.001
Total TAPES psychosocial adjustment	14	0	55.00	1.627	55.00	6.09	45	70	0.980	0.597	0.930	0.308
Total TAPES Prostheses Satisfection	14	0	35.21	2.640	35.50	9.88	10	49	−1.045	0.597	0.917	0.198

## Data Availability

The data that support the findings of this study are available from the corresponding author upon reasonable request.

## Abbreviations

The following abbreviations are used in this manuscript:

LLA	Lower limb amputation
PAD	Peripheral artery disease
DM	Diabetes mellitus
EQ-VAS	EuroQol Visual Analogue Scale
GAD-7	Generalized Anxiety Disorder-7
PHQ-9	Patient Health Questionnaire-9
ASonA	Antecedents and Self-Efficacy on Adherence
BIS	Body Image Scale
TAPES	Trinity Amputation and Prosthesis Experience Scales

## Appendix A

**Table A1:** Key findings emerging from the triangulation analysis.

Convergence Category	Amputees Quantitative	Amputees Qualitative	Prosthesis Users Quantitative	Prosthesis Users Qualitative	Agreement
ACCEPTANCE AND ADJUSTMENT					
**Illness acceptance**	Most patients report having accepted their medical condition (ASonA_01: 2.50 ± 1.45; range: 0–4).	Most patients verbally express passive acceptance of their clinical condition.	Most patients report having accepted their medical condition (ASonA_01: 3.00 ± 1.24; range: 0–4).	Most patients verbally express passive acceptance of their clinical condition, although there is positivity related to the hope of functional recovery thanks to the prosthesis.	*Agreement: 1 × 3, 2 × 4 (=2)* *Partial agreement: 1 × 2, 1 × 4, 2 × 3, 3 × 4 (=4)*
**Adaptation to the prosthesis**	Silence	Silence	All patients report having adapted to the prosthesis (TAPES_01: 4.57 ± 0.51; range: 4–5).	Almost all patients report having adapted to the prosthesis, although several report physical fatigue and perceiving the prosthesis as a foreign body.	*Agreement: 3 × 4 (=1)* *Silence: 1 × 3; 1 × 4; 2 × 3; 2 × 4 (=4)* *Not applicable: 1 × 2 (=1)*
**Body image**	Several patients report feeling good about the physical changes resulting from their medical condition (BIS: 6.43 ± 6.90; range 0–21).	No difficulties regarding body image are reported, although some mention limb loss and phantom limb.	Several patients report feeling good about the physical changes resulting from their medical condition (BIS: 6.07 ± 8.56; range: 0–25).	Although most patients report feeling good with their prosthesis thanks to the functional recovery obtained, several express fear of others’ judgment and refer to the presence of phantom limb.	*Agreement: 1 × 3, 2 × 4 (=2)* *Partial agreement: 1 × 2, 1 × 4, 2 × 3, 3 × 4 (=4)*
ADHERENCE					
**Pharmacological adherence**	All patients report adhering to medical prescriptions (ASonA_08: 3.57 ± 0.51; range: 0–4).	Most patients say they do not adequately adhere to prescriptions due to forgetfulness and/or lack of perceived benefit.	All patients report adhering to medical prescriptions (ASonA_08: 3.57 ± 0.94; range: 0–4).	All patients express willingness to adhere to medical prescriptions and to perform the recommended physiotherapy to improve their health and gain greater well-being and autonomy.	*Agreement: 1 × 3; 1 × 4; 3 × 4 (=3)* *Disagreement: 1 × 2; 2 × 3; 2 × 4 (=3)*
**Behavioral adherence**	All patients report following a healthy lifestyle (ASonA_09: 3.05 ± 1.35; range: 0–4).	Most patients say they do not follow a healthy lifestyle, not limiting food and consuming alcohol or sugary drinks.	All patients report following a healthy lifestyle (ASonA_09: 3.05 ± 1.62; range: 0–4).	All patients express the desire to improve their eating habits and adopt a healthy lifestyle.	*Agreement: 1 × 3; 1 × 4; 3 × 4 (=3)* *Disagreement: 1 × 2; 2 × 3; 2 × 4 (=3)*
EMOTIONAL EXPERIENCE					
**Anxiety**	The sample reports a mild level of anxiety (GAD-07: 4.71 ± 5.40; range: 0–21).	Most patients report moderate anxiety symptoms, mainly related to worries about the future.	The sample reports a mild level of anxiety (GAD-07: 4.29 ± 5.81; range: 0–21).	Although some patients report concerns about returning home, most show positivity, motivation, and hope for recovery.	*Agreement: 1 × 3 (=1)* *Partial agreement: 1 × 4, 3 × 4 (=2)* *Disagreement: 1 × 2; 2 × 3, 2 × 4 (=3)*
**Depression**	The entire sample reports a subthreshold level of depression (PHQ-9: 4.07 ± 4.60; range: 0–27).	Most patients report marked low mood due to disappointment about the diagnosis and loss of their health.	The entire sample reports a subthreshold level of depression (PHQ-9: 3.57 ± 4.01; range: 0–27).	Most patients report an improvement in mood following adaptation to the prosthesis and the benefits derived from it.	*Agreement: 1 × 3 (=1)* *Partial agreement: 1 × 4, 3 × 4 (=2)* *Disagreement: 1 × 2, 2 × 3, 2 × 4 (=3)*
**Quality of life**	Most patients perceive themselves to be in good health and to have good quality of life (EQ-VAS: 66.79 ± 21.89; range 20–100).	Almost all patients report experiencing a worsening in their quality of life due to loss of autonomy and increased difficulty performing daily activities.	Most patients perceive themselves to be in good health and to have good quality of life (EQ-VAS: 78.85 ± 17.10; range 50–100).	Almost all patients report an improvement in quality of life thanks to regained autonomy and partial independence.	*Agreement: 1 × 3, 1 × 4, 3 × 4 (=3)* *Disagreement: 1 × 2, 2 × 3, 2 × 4 (=3)*
**Denial**	Silence	Almost all patients show emotional detachment and denial of the event, both of the disease diagnosis and the limb amputation.	Silence	Most patients show emotional distance and denial of the impact of their clinical condition, especially regarding the diabetes diagnosis.	*Agreement: 2 × 4 (=1)* *Silence: 1 × 2, 1 × 4, 2 × 3, 3 × 4 (=4)* *Not applicable: 1 × 3 (=1)*
**Optimism**	Most patients consider themselves optimistic about their health (AsonA_10h: 2.57 ± 1.50; range 0–4).	Several patients report positive future expectations about their clinical and functional recovery after amputation.	Most patients consider themselves optimistic about their health (AsonA_10h: 3.00 ± 1.35; range 0–4).	Several patients report being positive about regaining their autonomy and progress with the prosthesis.	*Agreement: 1 × 2; 1 × 3; 1 × 4, 2 × 3; 2 × 4; 3 × 4 (=6)*
PERCEIVED SUPPORT					
**Family**	Almost all patients report receiving good support from their family (ASonA_03: 3.43 ± 0.85; range: 0–4).	Several patients report receiving excellent support from their family.	Almost all patients report receiving good support from their family (ASonA_03: 3.21 ± 1.42; range: 0–4).	Almost all patients report receiving excellent support from their family.	*Agreement: 1 × 2; 1 × 3; 1 × 4, 2 × 3; 2 × 4; 3 × 4 (=6)*
**Social support**	Several patients report receiving fair support from their network of friends (ASonA_04: 1.86 ± 1.46; range: 0–4).	Silence	Several patients report receiving adequate support from their network of friends (ASonA_04: 2.50 ± 1.87; range: 0–4).	Some patients report receiving support from their social network.	*Agreement: 1 × 3; 1 × 4; 3 × 4 (=3)* *Silence: 1 × 2; 2 × 3; 2 × 4 (=3)*
					***Total (=72)*** *Agreement (n = 32)* *Partial Agreement (n = 12)* *Disagreement (n = 15)* *Silence (n = 11)* *Not Applicable (n = 2)*
